# Sexuality in Ultra-High Risk for Psychosis and First-Episode Psychosis. A Systematic Review of Literature

**DOI:** 10.3389/fpsyt.2021.750033

**Published:** 2021-10-27

**Authors:** Giacomo Ciocca, Tommaso B. Jannini, Michele Ribolsi, Rodolfo Rossi, Cinzia Niolu, Alberto Siracusano, Emmanuele A. Jannini, Giorgio Di Lorenzo

**Affiliations:** ^1^Department of Dynamic and Clinical Psychology, and Health Studies, Sapienza University of Rome, Rome, Italy; ^2^Department of Systems Medicine, University of Rome Tor Vergata, Rome, Italy; ^3^Unit of Neurology, Neurobiology, Neurophysiology and Psychiatry, Department of Medicine, Campus Bio-Medico University, Rome, Italy; ^4^IRCCS – Fondazione Santa Lucia, Rome, Italy

**Keywords:** ultra-high risk for psychosis, first-episode psychosis, sexuality, mental health promotion, sexual trauma

## Abstract

A considerable body of literature reports that individuals with psychotic disorders often suffer from sexual dysfunctions (SDs), with these representing a major unmet need. Long-term antipsychotic drug treatment may be the main cause for SDs in psychotic patients, through a plethora of different mechanisms, including prolactin dyscrasia, histamine-mediated sedation, and serotonin-induced sexual demotivation. However, a few pieces of evidence treat sexuality in patients at risk or the onset of psychosis. For this purpose, we systematically reviewed literature of the last 10 years in order to investigate sexuality in ultra-high risk (UHR) for psychosis and first-episode psychosis (FEP). We included in our review 34 articles fitting our research criteria on SDs in UHR and FEP. Evidence of SDs in the transition from UHR to FEP emerges through the selected studies. In FEP, sexuality is affected by the severity of the psychotic symptoms and, in some cases, by the iatrogenic effects of psychopharmacological treatment. Further experimental and clinical studies should systematically investigate the role of sexual functioning in the transition from UHR to FEP and, consequently, clarify whether or not SDs could be considered a possible marker for the onset of psychosis in at-risk populations. Moreover, psychiatrists and clinical psychologists should take into consideration the role of sexual life in young people with prodromal mental symptoms or at the onset of psychosis. Focusing on a thorough sexual evaluation might be a major challenge that could break down barriers of mental health promotion among young people with schizophrenia-spectrum disorders and therefore achieve better clinical outcomes.

## Introduction

Psychotic spectrum disorders (PSDs), e.g., schizophrenia. schizoaffective disorder, affective disorders with psychotic manifestations, are devastating conditions generally accompanied by a consistent number of medical comorbidities (1).

It is well-established in literature that individuals with psychiatric disorders, psychosis in particular, can often suffer from sexual dysfunctions (SDs) (2). Indeed, a major cause of impairment may be the patients' long-term drug treatment, which affects sexuality through a plethora of different mechanisms, including prolactin dyscrasia, histamine-mediated sedation, and serotonin-induced sexual demotivation (3, 4). Sexual function (SF) can be impaired in many domains, ranging from desire, arousal, erection, and ejaculation in males, to orgasm (5, 6). Therefore, it is easy to understand how the prevalence of sexual dysfunctions in psychotic patients is much higher than in the general population (7).

First-episode psychosis (FEP) is defined as the first time a person experiences psychosis or psychotic symptoms. Patients with FEP go through different periods of transition, with phases of well-being alternating with prodromal states characterized by (i) non-psychotic behavioral disorders (such as depression or obsessive-compulsive symptoms); (ii) attenuated psychotic symptoms that do not require treatment; (iii) psychotic symptoms that require initial antipsychotic drug therapy; (iv) a formal diagnosis of the schizophreniform disorder and, subsequently, of schizophrenia (8).

The trajectory to developing first-episode psychosis was systematized in the last years by the concept of ultra-high risk (UHR), a sub-clinical period characterized by one of the following conditions and criteria: the attenuated psychotic symptom (APS) criterion, the brief limited intermittent psychotic symptom (BLIPS) criterion, and the genetic risk and functional decline (GRFD) criterion (9). Clinicians and researchers consider this period and the presence of UHR criteria as a possible phase to prevent a possible imminent frank psychosis (10).

Within the context of these clinical scenarios, it is useful to understand the role of sexuality. Sexuality itself has a large number of meanings, namely symbolizations, perceptions, impulses. Sexuality can easily fit inside a delusional, erotomaniac, or persecutory frame (11). On the other hand, a recent and relevant overview article pointed out the importance of considering sexuality in the psychiatric field and severe mental illnesses (12).

It is well-established in literature how people with psychotic spectrum disorders have serious impairments in the theory of mind (ToM) domain (13). ToM refers to the mental ability, defined as mentalization, to understand other people's behavior, considering it the result of different mental states. Since interpersonal relationships and intimacy play a key role in early psychosexual development, it is easy to imagine how mentalization among these patients might be crucial during these years. A recent review article has indeed underscored how dating might be extremely challenging for people with psychotic disorders, as romantic and intimate relationships are often precluded for such patients (14). From a psychodynamic point of view, a disrupted ToM might result in a compromised capacity to integrate sexual states in one's own developing identity, therefore producing high levels of distress (15). To this end, it is not surprising that early evidence has indeed shown how a poor premorbid sociosexual functioning is associated with a greater severity of negative symptoms as well as current social withdrawal (16).

UHR and FEP are critical moments in the clinical history of psychosis. During these times, sexuality is indeed completely reworked in its symbolic-relational connotations, characterized by remarkable impairments, and delusional contents. In addition, it also remains a major unmet need in the life of psychotic patients. Some interesting studies have indeed found that clinicians often fail to evaluate sexual problems, with this having dramatic repercussions on the partner, on the couple, on the therapy itself, and eventually on the overall quality of life (QOL) (17). In a recent article, the Authors surveyed a group of 750 psychiatrists, investigating their clinical care algorithms; the findings suggested how little importance is generally given to sexual health (18). In particular, only up to 3% of clinicians reported conduct a thorough sexual assessment on people with severe mental illnesses.

Since sexual dysfunction deeply weighs on QOL (19), it is easy to understand how neglecting sexual health might represent a relevant barrier in mental health promotion and a dangerous risk factor among young people with a clinical high risk of developing full-blown schizophrenia. In keeping more closely with this issue, two recent review articles discuss how important it is to better assess intimacy and sexuality among young people with FEP and psychotic disorders in general (14, 20). However, to date, a comprehensive review of sexual dysfunction among patients with a clinical high risk of developing psychosis is still missing.

Considering that sexual health is a perfect crossover between mental and physical health and yet a major unmet need among people with UHR and FEP, the aim of this present article is to enhance awareness on this important topic. For this purpose, we reviewed the most relevant original experimental articles found in literature on the relationship between sexuality and psychosis in UHR and FEP individuals.

## Methods

### Search Strategies

For this review, a thorough analysis was conducted of literature focused on Sexual functioning, First Episode Psychosis, and Ultra-High Risk for psychosis.

A computerized search was performed to identify a full relevant experimental article in PubMed, Web of Science, and Scopus on sexual functioning in First-Episode Psychosis and Ultra-High Risk for psychosis, published from January 2010 up to December 2020.

The following search terms were used: “First Episode Psychosis” AND “Sexuality” OR “First Episode Psychosis” AND “Sexual Dysfunction” OR “First Episode Psychosis” AND “Sexual Behavior” OR “Ultra-High Risk for Psychosis” AND “Sexuality” OR “Ultra-High Risk for psychosis” AND “Sexual Dysfunction” OR “Ultra-High Risk for psychosis” AND “Sexual Behavior.”

Inclusion criteria were: English published studies with a high/medium level of evidence according to the Canadian Task Force on the Periodic Health Examination (21), i.e., randomized controlled trials (RCTs), case-control, and cross-sectional studies; the articles must include patients diagnosed with either FEP or UHR for psychosis according to the DSM-V (1) or ICD-10/ICD-11 (22).

**Figure 1:**
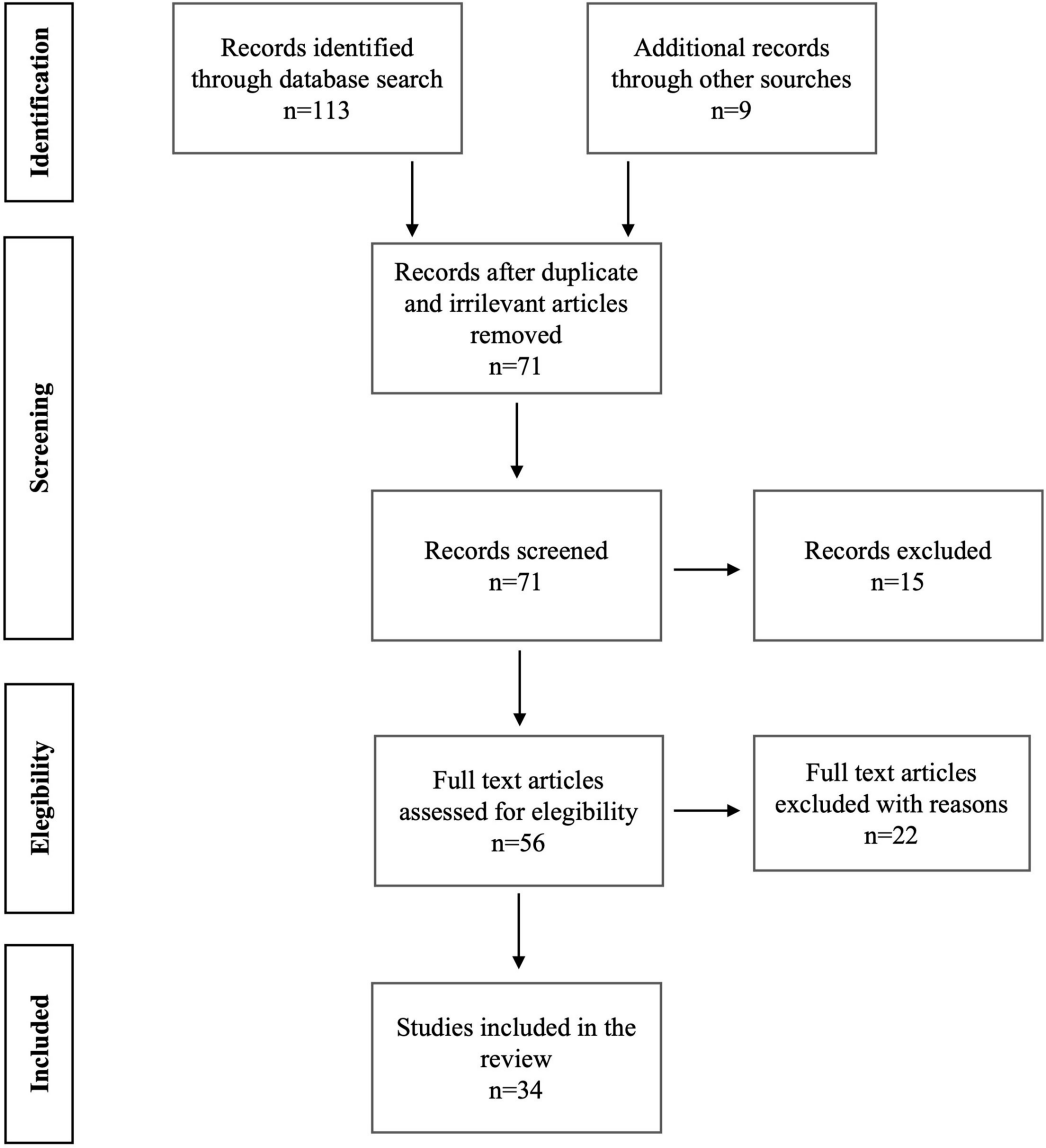
Prisma figure shows the search strategies performed in Pubmed, Scopus, and Web of Science. It also reports the literature results we found.

Exclusion criteria were review articles, books/book chapters, editorials, theses; studies concerning characteristics of personality not connected to sexuality; chemical, biological, and other field studies different from sexology and sexual medicine. Figure 1 represents the flowchart showing further details on the literature search and the selection of articles included in this literature review.

## Results

A total of 113 articles were retrieved on PubMed, Scopus, and Web of Science. Subsequently, 47 articles were selected based on their abstracts. Finally, 34 articles, of which 1 was a RCT, 7 were retrospective studies and 26 were cross-sectional studies, fully satisfied the criteria for the review (Figure 1, Table 1). We broke down these literature results between UHR and FEP into the following paragraphs.

**Table 1:** Studies inserted in the review analysis about the sexuality in ultra-high risk for psychosis and first-episode psychosis.

**References**	**Psychiatric condition**	**Design of the study**	**Sample size**	**Tools and measures**		**Main findings**
				***Sexual behavior measures***	**Other psychiatric measures**	
Adan Sanchez et al. (23)	UHR	Cross-sectional	103	Items relating to sexual risk behavior	Brief Psychiatric Rating Scale (BPRS)	Young people with mental disorders reported major sexual risk behaviors and also a relevant frequency of sexually transmitted infections (STIs). The contraceptive methods use are few, and also the most of pregnancy is unplanned.
Appiah-Kusi et al. (24)	UHR	Case-control	68	N/A	Childhood Trauma Questionnaire (CTQ); Beck Depression Inventory (BDI); State-Trait Anxiety Inventory (STAI); Cannabis Experience Questionnaire (CEQ); Paranoid Thought Scales (PTS); Psychosis Screening Questionnaire (PSQ)	The 21% of UHR patients assessed had been exposed to sexual abuse. On the whole, this study confirmed the pathogenetic role of childhood trauma in the development of psychosis. Moreover, major negative self-schemas were found in UHR patients compared to healthy controls.
Bechdolf et al. (25)	UHR	Cross-sectional	92		Comprehensive Assessment of At-Risk Mental States (CAARMS); General Trauma Questionnaire (GTQ)	UHR patients with past sexual trauma have a major risk to develop psychosis. About one-quarter of UHR patients had a history of sexual abuse. Sexual trauma in the UHR population is, therefore, a predictive factor for the conversion in FEP and frank psychosis.
Dragt et al. (26)	UHR	Cross-sectional	72	N/A	Basic Data Form (BDF); Client Service Receipt Inventory (CSRI); Premorbid Adjustment Scale (PAS)	Social–sexual aspects together with the social–personal adjustment were the best predictors for developing a first psychotic episode in the assessed UHR group.
Egerton et al. (27)	UHR	Case-control	67	Childhood Experience of Care and Abuse Questionnaire (CECA-Q)	Comprehensive Assessment of At-Risk Mental States (CAARMS); Positive and Negative Syndrome Scale (PANSS); Hamilton Depression and Anxiety Rating Scales; Positron Emission Tomography (PET).	Among childhood adversities, only sexual and physical abuse was found with a higher prevalence in the UHR group compared to the healthy control. Subjects with experiences of physical or sexual abuse in childhood show higher dopamine function in the associative striatum.
Marques et al. (28)	UHR	Case-control	124	Sexual Function Questionnaire (SFQ)	Positive and Negative Syndrome Scale (PANSS); Calgary Depression Scale	UHR people then converted into psychotic patients primarily revealed a great sexual impairment, as sexual dysfunctions, compared to UHR individuals no developed psychosis.
Rapado-Castro et al. (29)	UHR	Cross-sectional	62	N/A	Comprehensive Assessment of At-Risk Mental States (CAARMS); Scale for the Assessment of Negative Symptoms (SANS); Brief Psychiatric Rating Scale (BPRS); Hamilton Rating Scale for Depression (HRSD); Global Assessment of functioning Scale (GAF); Quality of Life Scale (QLS); Adult Intelligence Scale-Revised (WAIS-R); Wechsler Abbreviated Scale of Intelligence (WASI); Intelligence Scale for Children (WISC-III); Childhood Trauma Questionnaire (CTQ); magnetic resonance imaging (MRI)	UHR patients with experiences of sexual abuse show reductions in prefrontal and temporal brain regions, highlighting a particular morphological vulnerability of superior prefrontal and temporal structures to sexual trauma. Sexual abuse, inducing a reduction of cortical thickness in the right middle temporal gyrus, has an indirect effect on the transition to psychosis.
Sahin et al. (30)	UHR	Case-control	193	N/A	Brief Psychiatric Rating Scale (BPRS); Scale for the Assessment of Negative Symptoms (SANS); Scale for the Assessment of Positive Symptoms (SAPS)	In UHR females the scores for the sexual abuse of CTQ were significantly higher than in males and in the overall group. The sexual abuse was related to positive symptoms. Moreover, the UHR group
					Trauma Questionnaire (CTQ); Calgary Depression Scale for Schizophrenia (CDSS).	showed higher scores at sexual abuse compared to healthy controls. Overall, childhood trauma is a predictive factor related to both patients with UHR and first-episode schizophrenia.
Schmidt et al. (31)	UHR	Cross-sectional	73	N/A	Structured Interview for Psychosis-Risk Syndromes (SIPS); Trauma And Distress Scale (TADS); German Stress-Coping-Questionnaires; German Competence and Control Beliefs Questionnaire (FKK); Beck Depression Inventory (BDI-II); Mini International Neuropsychiatric Interview in its version for adults (MINI)-suicidality scale.	The 21.5% of CHR patients reported sexual abuse and this aspect is inserted in the relationship between traumatic experience and suicidality. Moreover, sexual abuse is correlated with the major use of dysfunctional coping strategies. On the whole, sexual abuse has resulted related to suicidal ideation in CHR patients.
Thompson et al. (32)	UHR	Cross-sectional	93	N/A	Comprehensive Assessment of At-Risk Mental States (CAARMS)	Being watched in the shower/toilet or undressing were more frequent sexual delusions relived in UHR people. These delusion types in UHR are often related to a history of sexual trauma.
Thompson et al. (33)	UHR	Cross-sectional	416 (233)	N/A	Childhood Trauma Questionnaire (CTQ); Comprehensive Assessment of At-Risk Mental States (CAARMS); Brief Psychiatric Rating Scale (BPRS); Global Assessment of Functioning (GAF); Schedule for the Assessment of Negative Symptoms; Hamilton Rating Scale for Depression; Quality of Life Scale.	Females with UHR showed a higher score to CTQ for sexual abuse and physical abuse and in the overall sample the sexual abuse is related to the transition to psychosis. Therefore, past sexual trauma is the main risk factor to develop a frank psychosis in the UHR population, suggesting clinicians to investigate the sexual abuse experiences in people at risk of severe mental disorders.
Üçok et al. (34)	UHR	Cross-sectional	53	N/A	Brief Psychiatric Rating Scale (BPRS); Global Assessment of Functioning (GAF); Scale for the Assessment of Negative Symptoms (SANS); the Scale for the Assessment of Positive Symptoms (SAPS); the Childhood Trauma Questionnaire (CTQ); the Calgary Depression Scale for Schizophrenia (CDSS) and neuropsychological tests.	Patients with a history of physical trauma performed worse in attention, interference inhibition, working memory, and cognitive flexibility tests than individuals without physical trauma. However, no differences between subjects with and without a history of childhood emotional/sexual trauma were found in terms of cognitive performance
Velthorst et al. (35)	UHR	Case-control	127	N/A	Comprehensive Assessment of At-Risk Mental State (CAARMS).	A major association between female gender and sexual abuse was found in UHR patients. Moreover, sexual abuse was related to post-traumatic stress disorder symptoms and perceptual disturbance with sexual contents.
Ajnakina et al. (36)	FEP	Retrospective study	236	Childhood Experience of Care and Abuse Questionnaire (CECA-Q)	Positive and Negative Syndrome Scale (PANSS)	Childhood sexual abuse, physical abuse, and parental separation had a significant association with positive dimensions.
Bendall et al. (37)	FEP	Cross-sectional	28	Childhood Trauma Questionnaire (CTQ)	Positive and Negative Syndrome Scale (PANSS); Impact of Events Scale-Revised (IES-R); National Adult Reading Test (NART)	FEP patients who suffered a sexual abuse presented more positive and depressive symptoms, more severe delusions and hallucinations compared to their counterparts
Braehler et al. (38)	FEP	Cross-sectional	171	Childhood Trauma Questionnaire (CTQ);	Dissociative Experiences Scale (DES)	Severe childhood trauma is associated with greater dissociative symptoms, with emotional abuse showing the greatest association with dissociation
Brown et al. (39)	FEP	Cross-sectional	115	Broad indicators of sexual risk behavior were either developed or adapted from Visser and Smith (40)	Brief Psychiatric Rating Scale, Expanded Version (BPRS-E)	Increased frequency of unprotected sex in people with FEP suggests that those with psychosis are at increased STI risk and have distinct needs
Brown et al. (41)	FEP	Cross-sectional	112	Patient-reported questionnaire on sexual behavior	Kessler 10 Psychological Distress Scale; Rosenberg Self-Esteem Scale; Multidimensional Scale of Perceived Social Support; Opiate Treatment Index	Increased probability of inconsistent condom use was associated with clinical status, younger age, unemployment, and the absence of peer support for condom use.
Brown et al. (42)	FEP	Cross-sectional	112	Patient-reported questionnaire on sexual behavior;	Kessler 10 Psychological Distress Scale; Rosenberg Self-Esteem Scale; Multidimensional Scale of Perceived Social Support; Opiate Treatment Index;	Inconsistent condom use was predicted by clinical status, unemployment, and the absence of peer support for condom use.
Conus et al. (43)	FEP	Case-control	658	N/A	Early Psychosis File Questionnaire; EPFQ	
Ciocca et al. (44)	FEP	Cross-sectional	77	Udvalg for Kliniske Undersøgelser (UKU);	Positive and Negative Syndrome Scale (PANSS)	Correlation between sexual dysfunctions and psychopathology did not reveal any association in males. On the other hand, in females, general psychopathology and positive symptoms were linked to the alteration in vaginal lubrication.
Ciufolini et al. (45)	FEP	Cross-sectional	302	Childhood Experience of Care and Abuse Questionnaire (CECA-Q); cortisol saliva levels	N/A	Divergent effect of severe childhood abuse on HPA axis activity in patients with FEP and controls, with FEP showing a reduced cortisol awakening response and a less reactive HPA axis.
Del Cacho et al. (46)	FEP	Cross-sectional	118	Changes in Sexual Function Questionnaire (CSFQ); hormone blood levels	Positive and Negative Syndrome Scale (PANSS)	Evidence of better sexual function in healthy controls compared to patients with FEP. No association between both prolactin and testosterone and sexual function.
El Sayed El Taweel et al. (47)	FEP	Cross-sectional	50	Arizona Sexual Experience Scale (ASEX); designed questionnaire	Mini International Neuropsychiatric Interview (MINI); Positive and Negative Syndrome Scale (PANSS); the Montgomery–Asberg Depression Scale (MADRS)	Male patients with FEP reported significantly more sexual dysfunctions compared to controls. A statistically significant correlation was seen between the long duration of untreated psychosis, the severity of negative symptoms, and depressive symptoms with the severity of sexual dysfunction among patients with schizophrenia.
Gaber et al. (48)	FEP	Cross-sectional	80	International Index of Erectile Function (IIEF-5)	N/A	Males with FEP are more likely to develop erectile dysfunction (ED); the duration of untreated psychosis also leads a higher incidence of ED and hyperprolactinemia.
Hui et al. (49)	FEP	Cross-sectional	343	Udvalg for Kliniske Undersøgelser (UKU);	Scale for the Assessment of Positive Symptoms (SAPS); Scale for the Assessment of Negative Symptoms (SANS); Role Functioning Scale (RFS);	Sexual dysfunction was significantly and independently associated with being married, more general side effects, poorer functioning, and higher monthly income
Malik et al. (50)	FEP	Randomized controlled trial	498	Udvalg for Kliniske Undersugelser (UKU)	Positive and Negative Syndrome Scale (PANSS); St Hans Rating Scale	Only mild differences in sexual dysfunctions were seen among five antipsychotics groups, underscoring the role of the disease itself on the onset of these adverse effects.
Misiak et al. (51)	FEP	Cross-sectional	141	Childhood Experience of Care and Abuse Questionnaire (CECA-Q)	Positive and Negative Syndrome Scale (PANSS); the Montgomery–Asberg Depression Rating Scale (MADRS); the Young Mania Rating Scale (YMRS); the Global Assessment of Functioning (GAF)	FKBP5 gene methylation is significantly lower in FEP compared to controls. In particular, patients with parental apathy and sex abuse had significantly lower levels of FKBP5 methylation.
Ravichandran et al. (52)	Drug-naïve patients with psychosis	Cross-sectional	100	International Index of Erectile Functioning (IIEF)	Positive and Negative Syndrome Scale (PANSS)	Sexual dysfunction may be found in patients with psychotic disorders before starting their pharmacotherapy.
Reininghaus et al. (53)	FEP	Cross-sectional	147	N/A	Experience sampling method (ESM)	Elevated sensitivity to minor stressful events and enhanced threat anticipation are associated with more psychotic experiences.
Theleritis et al. (54)	FEP	Cross-sectional	239	Childhood Experience of Care and Abuse Questionnaire (CECA-Q); Brain-derived Neurotrophic Factor (BDNF) plasmatic levels	N/A	Significant effect of separation, physical, and sexual abuse on Brain-derived Neurotrophic Factor (BDNF), with patients with a history of sexual trauma showing lower plasmatic levels of Brain-derived Neurotrophic Factor (BDNF).
Theleritis et al. (55)	FEP	Cross-sectional	116	Sexual Function Questionnaire (SFQ); weight; BMI; waist; waist-hip ratio; hormone blood levels	Positive and Negative Syndrome Scale (PANSS); Calgary Depression Score (CDS)	Results showed a correlation of higher SFQ and higher BMI, leptin levels, waist-hip ratio, and lower testosterone levels
Tomassi et al. (56)	FEP	Cross-sectional	345	Childhood Experience of Care and Abuse Questionnaire (CECA-Q)	Cannabis Experiences Questionnaire (CEQ)	Sexual abuse was associated with a diagnosis of affective psychosis and higher rates of cannabis and heroin abuse; physical abuse was associated with lifetime use of heroin.
van Bruggen et al. (57)	FEP	Cross-sectional	40	Questionnaire for Sexual Dysfunction (QSD); hormone blood levels	Positive and Negative Syndrome Scale (PANSS)	Patients reported a significantly higher frequency of problems with ejaculation and problems with insensibility of genitals compared to controls. However, no relation between medication, hormonal levels, frequency of sexual activity, overall satisfaction with sexuality, and any of all sexual dysfunctions was found.

### Ultra-High Risk for Psychosis and Sexuality

Traditionally, sexual dysfunction in psychotic illnesses has always been linked to adverse side effects of psychopharmacology. However, sexuality can be severely impaired by numerous factors regardless of pharmacotherapy, like premorbid personality, i.e., schizoid or schizotypal, psychopathology itself, as a result of the negative effect of psychosis on personal and sexual relationships, and medical comorbidities that frequently occur in these patients (58). Indeed, a proof of concept of this was provided by a previous study where sexual function in UHR people was compared with healthy control subjects and with first-episode psychosis patients (28). The Authors demonstrated that UHR people that subsequently became full-blown psychotic patients primarily revealed greater sexual impairment compared to UHR individuals who did not develop psychosis. This evidence was not related to the gender or to the side effects of any antipsychotic treatment, as the UHR people were not undergoing any psychopharmacological therapy (28). In light of this study, it is possible to consider sexual dysfunctions as manifestations of UHR criteria-related psychotic symptoms. Moreover, it is also interesting to notice how the sexual content of thinking may be a direct consequence of early-life sexual abuse among young people with clinical high risk for psychosis.

#### UHR and Sexual Trauma

Although it is well-established how early trauma represents a vulnerability factor toward psychosis (59), previous research has also pointed out how the sexual nature of delusion demonstrates a close relationship with a past episode of sexual trauma or sexual abuse. For instance, being watched in the shower/toilet or undressing were the most frequent sexual delusions to be reported in UHR people (32). Generally, the relationship between sexual trauma and the UHR condition is mostly characterized by perceptual disturbances with sexual contents (35), symptomatologic elements that reveal once again to be predictive for psychosis.

In this regard, most of the experimental articles we reviewed affirmed that sexual abuse, physical abuse, and sexual trauma in childhood represent the main predictors for developing a frank psychosis in adulthood. More than 20% of people with UHR for psychosis had an experience of sexual abuse during childhood (24, 25, 31). Consistent with the literature, this dramatic evidence is mostly related both to the female gender and to higher levels of childhood trauma (33, 35).

Young individuals with at risk mental states who suffered from sexual abuse are also reported to have a higher severity and frequency of psychotic experiences (30, 53).

Moreover, recent evidence reported that UHR patients with a past clinical history of sexual abuse feature both cognitive and neuroanatomical impairments. In particular, patients with a history of physical trauma performed worse in attention, interference inhibition, working memory, and cognitive flexibility. Significant cortical thickness reductions in prefrontal and temporal brain regions were also observed in individuals at UHR for psychosis who reported moderate to high levels of childhood sexual abuse (29, 34). In addition, a study found that the striatal dopamine function, whose system is well-known to be impaired in PSDs, is increased in people with experiences of sexual abuse (27).

Therefore, it is possible to hypothesize that sexual trauma in childhood might represent a *primum movens* in people with UHR, with a devastating impact on the neuroanatomical and neurophysiological development.

### First Episode Psychosis and Sexuality

The overall prevalence of sexual impairments in an FEP study population may vary from 13 to 64%, mostly depending on how patients were both selected and assessed (47, 49, 52). The European First Episode Schizophrenia Trial (EUFEST) reported dysfunctions in many domains of sexuality, i.e., orgasm (15%), erectile dysfunction (17%), and decreased libido (30.8%) in patients that were randomly assigned to various antipsychotics. Findings also showed that the main side effects caused by the increase in prolactin resulted in amenorrhea, galactorrhea, and gynecomastia (50).

As these patients are often young, it is important to investigate sexual health during the psychiatric evaluation (39). For instance, people with psychiatric disorders are more prone to engage in risky sexual behaviors, with psychotic patients ranking among the top positions (60). Notably, young people with FEP are more likely to have unprotected sexual intercourse compared to their peers. Previous research highlighted how younger age, absence of peer support, unemployment, and clinical status were associated with an increased probability of condom misuse in these patients (41). Sexual knowledge among such patients is poor, not only from a physical and technical standpoint (i.e., how to perform sexual intercourse or other sexual practices) but also in terms of prevention of sexually transmissible infections (STIs) (61).

Hence, it is easy to understand how people with FEP are at greater risk of contracting STIs, therefore needing significant prevention strategies and risk-reduction measures (42).

#### FEP and Sexual Trauma

It is not surprising to see that, similarly to UHR patients, consistent evidence of sexual abuse during childhood among people with FEP is also present in literature. A recent systematic review has indeed pointed out how its prevalence spans from 6 to 40%, with women suffering more sexual abuse compared to men (62). Moreover, the impact of childhood trauma tends to be greater on later-stage symptoms. For instance, FEP patients who suffered sexual abuse showed more positive and depressive symptoms as well as more severe delusions and hallucinations compared to their counterparts (37). Consistent with this, sexually abused patients with FEP show poor premorbid functioning, longer DUP, fewer years of education, past history of suicide attempts, and other psychiatric disorders; moreover, they are more likely to be diagnosed with a lifetime substance use disorder (43).

This evidence is backed up by an interesting model, known as the *traumagenic neurodevelopmental model of psychosis* (TN), which links childhood sexual maltreatments in people with FEP to functional and structural brain alterations during young adulthood (63). TN posits how hypothalamic-pituitary-adrenal axis (HPA) alterations, structural cerebral changes, such as frontal lobe and hippocampus abnormalities, dopamine system impairments, and psychiatric comorbidities are consistently more frequent in FEP patients who suffered from sexual trauma. For instance, sexual abuse exerts a divergent effect on HPA axis activity in patients with FEP and controls, with FEP showing a reduced cortisol awakening response and a less reactive HPA axis (45). Sexually maltreated people with FEP also reported higher levels of dissociative symptoms compared to controls (38). To this end, childhood sexual abuse, physical abuse, and parental separation are reported to be significantly associated with higher positive and negative symptoms (30, 36), more intense psychotic experiences (53), more frequent diagnosis of affective psychosis and higher rates of cannabis and heroin abuse (56) among young patients with FEP. Interestingly, recent evidence reported how sexual trauma may also impact at a molecular and genetic level, with sexually abused FEP young individuals showing a lower methylation of the FKBP5 gene (51) and lower *brain-derived neurotrophic factor* (BDNF) plasmatic levels (54).

lower brain-derived neurotrophic factor (BDNF) levelsŤ]

**Figure 2:**
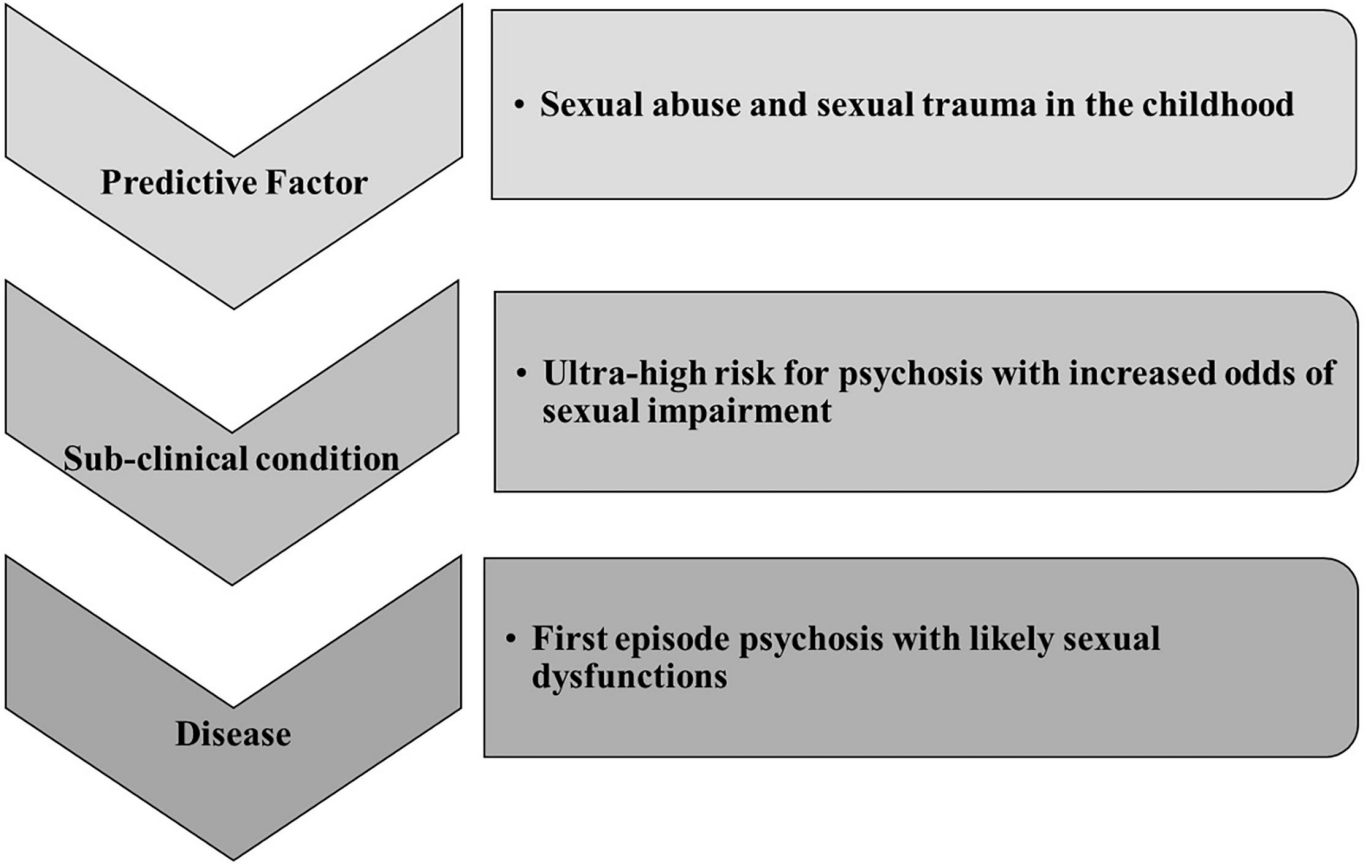
Our literature review is summarized in this figure showing the sexological trajectory of psychosis. It is based on literature finding mainly focused on sexual abuses experience. Sexual trauma can be considered a central etiological factor for UHR, FEP, and then psychosis. However, major clinical and research attention should be posed to sexual impairment during the UHR phase.

These findings underline and confirm what is extensively reported in literature; i.e., that early trauma deeply impacts on the development and severity of psychosis (64) (Figure 2).

#### FEP and Gender

If in recent years psychopathology has focused more on what is generically defined as gender medicine, in the case of sexological evaluation in patients with FEP a *genderized* approach takes on clinical and theoretical relevance (65). For example, literature suggests that men show more negative and obsessive-compulsive symptoms, are more prone to drug abuse and develop symptoms earlier than women (66). To date, however, only a few studies offered a dichotomous perspective on gender differences of sexual dysfunctions in FEP (44, 46, 55, 65–67). Apart from the prevalence, which is slightly the same in both sexes, a conflicting result is found in two studies. In the first study, the authors described how vaginal lubrification and orgasm are significatively correlated with either general or positive PANSS scores, without finding any similar homolog association among male subjects (44). On the other hand, the second study reported that sexual functioning was negatively correlated with disorganized and positive symptoms in males and with higher scores in negative symptoms among females (46). Finally, a study on SF and metabolic structure among men found a correlation between higher Sexual Function Questionnaire scores (i.e., worse sexual outcome) and BMI, leptin levels, and waist-hip ratio (55). This highlights how obesity could play a mediating role between psychopathology and sexual dysfunction, with hormonal alterations in testosterone levels in young male patients with psychosis. A recent study evaluated 40 male subjects and found that men with FEP have a higher probability of developing erectile dysfunction (ED), with a DUP increasing the risk of both ED and hyperprolactinemia (48). Women, on the other hand, have more resources and social support during the pre-crisis phase, with levels of social functioning playing a protective role, although they show more depressive, anxious, and affective symptoms (67). Although these findings are scarce and poorly consistent with each other, they highlight the importance of considering a gender-based perspective in sexual function among patients with FEP in order to obtain a better therapeutic outcome.

#### Psychopharmacotherapy and Pathophysiology in FEP

Unlike UHR, people with FEP have generally had their first encounter with a specialist. As they are most commonly under psychopharmacological therapy, sexual dysfunctions are certainly enhanced by adverse side effects of antipsychotics (28). Rates of sexual dysfunction recurrence are indeed higher in FEP people under psychopharmacotherapies. To this end, a recent study conducted on a very large sample, within the EUFEST protocol, investigated these aspects during the first year of drug treatment, with particular attention to hormonal alterations and sexual dysfunctions (50). The authors indicated that other than age and general PANSS score, prolactin levels in the blood also predicted erectile dysfunctions in males.

Consistent with this, another study on 243 patients diagnosed with FEP and treated with antipsychotics found a prevalence in sexual impairment of 46% (2). The authors also reported that risperidone weighed on sexuality in a dose-dependent manner, causing more severe sexual dysfunctions compared to other active principles. Moreover, at the univariate logistic regression, risperidone showed a 7-fold increased risk of developing SDs.

However, a growing body of evidence stresses the importance of the disease pathophysiology as a major cause of sexual impairment in drug-naïve patients with FEP. Multiple studies point out how impaired sexuality may be either predicted by or correlated to heavier psychotic symptomatology and longer DUP, without showing any association with hormones blood levels, like prolactin, or testosterone (46, 47, 50, 52), although hyperprolactinemia may be a clinical feature of drug-naïve people with FEP (68). Moreover, these results are consistent with and endorsed by evidence from FEP individuals treated either with olanzapine or risperidone (57). Although patients in the drug groups reported a higher prevalence of sexual dysfunctions (without any difference between olanzapine and risperidone) compared to controls, no relationship between medication, hormones, frequency of sexual activity, and overall sexual satisfaction was found.

## Discussion and Conclusions

When a physical, psychic, social, or relational problem arises, sexuality is almost always involved in its dysfunctional declination. Over time, a large body of literature has focused on the role of sexual dysfunction as a prodromal symptom of a possible psychotic onset (28). This omnipresent feature of sexuality during either a chronic or transient state of illness takes on considerable importance in young people. This is indeed testified by the families of FEP patients, whose major concerns for their offspring, along with substance abuse and self-esteem, is also sexuality and intimacy (69).

Similarly to major depression, in UHR individuals and people with FEP sexual dysfunctions may be considered as a prodrome or a consequence of either psychopathology or psychopharmacology.

Most of the early evidence indeed underscores a powerful intrinsic influence of psychosis itself on sexual functioning. Possible explanations for this might be found in the neurobiological mechanisms underpinning both sexual dysfunctions and schizophrenia. In a recent review article of five studies about the impact of psychosis on sexual functioning, Varga-Cáceres et al. hypothesized the role of a striatal dopaminergic dysregulation as a major cause of dysfunction in sexual life among unmedicated psychotic patients (20). Although the striatal dopaminergic pathway is key in reward-predicting cues, and therefore in sexual motivation (70), other possible explanations must be taken into account to better define the intricate relationship between these two conditions. In addition to dopamine and striatum, psychosis, and sexual disorders share in fact impairments both at other neurotransmitters and at different brain networks level. From a neurotransmitter perspective, we would like to focus on glutamate, oxytocin, and endocannabinoids. Firstly, it is well-established in literature that schizophrenia is characterized by high levels of glutamate in the prefrontal cortex (71). Interestingly, a recent article highlighted how glutamate afferents from the prefrontal cortex modulate the activation of the *nucleus accumbens*, a key hub for pleasure and reward, in female rodents during sexual intercourse (72). Keeping this in mind, we infer that impaired levels of glutamate in psychosis might be responsible for altered sexual functioning. Secondly, extensive data suggests that impairments in social life occurring in psychosis may also be explained by a dysfunctional oxytocinergic system, a key modulator of emotional, sexual, and social processes (73, 74). Disorders in ejaculation and consequently in orgasm may indeed be linked to a compromised dopamine and oxytocin pathways between the hypothalamus and the limbic structures (75). Thirdly, the endocannabinoid system (ECS) is considered a major modulator of the hedonic effects of natural rewards, such as sexual activity, with endogenous cannabinoids showing a significant relationship with sexual arousal (76, 77). As ECS has shown to be impaired in individuals with psychotic disorders, including those at the early stages of the illness without antipsychotic treatment (78), we might also hypothesize that altered levels of endocannabinoids might be another important etiology of sexual dysfunctions in psychosis.

From a brain network-based perspective, an altered mesocortical pathway may also be responsible for an impaired sexual drive in psychosis. The dorsolateral prefrontal cortex, an area involved in goal-directed behaviors and motivational significance to sexual stimuli (79), is mainly modulated by dopamine from the ventral tegmentum. As its functional alterations are largely reported in psychosis, it would explain why psychosis itself, featuring flattened affect, anhedonia, reduced social drive, and loss of motivation, may account for impaired sexual desire (80).

Finally, there have been several attempts to define psychosis as a disconnection syndrome. In the light of this, we might postulate that dysfunctions in the three major resting-state networks, i.e. default mode, salience, and central executive networks, which account for most of the behavioral, cognitive, and social deficits of psychosis, might also be an explanation for sexual deficits among people with psychotic disorders (81). In other words, as this model seems to explain the neural bases of intimacy and empathy, it might also be interpreted as a neurophysiological biotype for the comorbidity between psychosis and sexual dysfunctions.

On the other hand, extensive literature has grown on the fact that antipsychotics may impact sexual functioning, mostly because they affect the tuberoinfundibular pathway, therefore resulting in high levels of prolactinemia (82). For this reason, it is of vital importance to actively implement new therapeutical strategies to lower the incidence of these adverse effects. For instance, in addition to the well-known aripiprazole, whose switching (83) or augmentation (84) are now established among therapeutic options, new drugs are proving to decrease their impact on sexual functioning. In particular, both brexpiprazole (85) and the recently introduced SEP-363856 (86) have shown significantly low levels of prolactinemia in patients with schizophrenia.

After our screening of literature, several issues could be considered as lacking in the assessment and prevention of a possible mental disease. For example, a well-known phenomenon of compulsive masturbation related to psychiatric distress was not studied in light of the UHR or FEP condition (87), although it should be considered as one of the markers for the potential development of severe mental illness.

At the same time, hypersexuality and compulsive use of pornography could be indicators of psychosocial distress in more fragile personalities. Therefore, clinicians and researchers should take into consideration a possible relationship between these dysfunctional aspects of sexual behavior and a predisposition toward psychopathology, above all in young adults and adolescents (88, 89). Another understudied aspect, but likely central for psychologically-vulnerable males, is penile dysmorphia, a specific sexual symptom comparable to body dysmorphic disorder. If this symptom is often treated in the urological and andrological fields, with potential surgical approaches, a psychiatric assessment would be highly recommended when penile size becomes an obsession (90).

Hence, the behavioral spectrum related to the predictive role of sexual behavior toward psychopathology or dysfunctional condition is large and variegated. Literature mainly investigated sexual functioning and sexual history related to a traumatic experience in association with FEP and UHR, although other behavioral phenomena could be correlated to the exacerbation of psychopathology in youths. In this regard, clinical protocols should take into consideration more adequate assessment praxis along with the standardized psychometric procedure. To date, many psychometric tools are available to assess sexual behavior in all its facets, from sexual functioning to dysregulated and compulsive sexual behavior. These questionnaires should be integrated into psychodiagnostics in order to detect and possibly prevent a potential predictive association between sexual problems and possible mental disease.

Nevertheless, sexuality has to be considered as one of the major unmet needs and, likewise, sexual impairments as an important barrier to mental health promotion for young people with schizophrenia-spectrum diseases (91). Young people with UHR or FEP might be indeed facing what could be defined as a “*syndemics*.” Although this term is most specifically used within contexts of infectious diseases, syndemics refers to a biosocial model in which two or more medical conditions co-occur and interact leading to worse negative health outcomes (92). This model may also be applied to sexuality among young people with a clinical high risk of psychosis. Disease-related sexual dysfunctions, higher rates of sexual risky behavior, STIs, and sexual self-stigma, together with the well-known array of psychotic symptoms may, in fact, synergistically enhance feelings of worthlessness and increase social isolation (93). It is therefore easy to understand how this may dramatically affect an already delicate doctor-patient relationship, especially in a population of young individuals. Having such an unmet need might take away useful pathways of communication between the patient and the psychiatrist/clinical psychologist, therefore leading to worse clinical outcomes and higher rates of therapy drop-outs.

Thus, focusing on a thorough sexual evaluation might be a major challenge that could break down barriers of mental health promotion among young people with schizophrenia-spectrum diseases.

In conclusion, the clinician's defensive denial of a patient's sexuality can significantly affect the treatment, therefore hindering the achievement of a general well-being to which every therapeutic practice should aspire. With this in mind, psychiatry and clinical psychology should considerably include sexual assessment, as well as sexological expertise, in their research and clinical praxis.

Although this review benefits of a broad focus, our study suffers from a number of major limitations. Firstly, the vast majority of the studies were cross-sectional, with only one RCT. This means that no follow-up data was present; thus caution must be taken when generalizing these findings. Secondly, the high heterogeneity of studies did not allow to use a metanalytic approach, which would have been desirable in order to draw more consistent conclusions on this topic. Thirdly, none of the mentioned studies used a structural equation modeling approach, that would allow to gain information on the possible modulating or moderating effects of certain variables (i.e., gender, type of psychosis, *etc*.) on other variables (such as desire, arousal, orgasm, or overall sexual behavior). For this reason, with this article we aim to encourage future studies that will shed a brighter light on psychosis and sexual functioning.

## Author Contributions

GC: manuscript drafting and conceptualization. TBJ: investigation and manuscript drafting. MR and RR: literature review. CN, AS, and EAJ: data curation and literature review. GDL: supervision and project administration. All authors contributed to the article and approved the submitted version.

## Conflict of Interest

The authors declare that the research was conducted in the absence of any commercial or financial relationships that could be construed as a potential conflict of interest.

## Publisher's Note

All claims expressed in this article are solely those of the authors and do not necessarily represent those of their affiliated organizations, or those of the publisher, the editors and the reviewers. Any product that may be evaluated in this article, or claim that may be made by its manufacturer, is not guaranteed or endorsed by the publisher.
